# Arginase-II gene deficiency reduces skeletal muscle aging in mice

**DOI:** 10.18632/aging.206173

**Published:** 2024-12-12

**Authors:** Matteo Caretti, Duilio Michele Potenza, Guillaume Ajalbert, Urs Albrecht, Xiu-Fen Ming, Andrea Brenna, Zhihong Yang

**Affiliations:** 1 Department of Endocrinology, Metabolism, and Cardiovascular System, Faculty of Science and Medicine, University of Fribourg, Fribourg 1700, Switzerland; 2 Department of Biology, Faculty of Science and Medicine, University of Fribourg, Fribourg 1700, Switzerland

**Keywords:** aging, arginase-II, cellular senescence, fibrosis, physical activity, skeletal muscle

## Abstract

Age-associated sarcopenia decreases mobility and is promoted by cell senescence, inflammation, and fibrosis. The mitochondrial enzyme arginase-II (Arg-II) plays a causal role in aging and age-associated diseases. Therefore, we aim to explore the role of Arg-II in age-associated decline of physical activity and skeletal muscle aging in a mouse model. Young (4–6 months) and old (20–24 months) wild-type (*wt*) mice and mice deficient in *arg-ii* (*arg-ii^-/-^*) of both sexes are investigated. We demonstrate a decreased physical performance of old *wt* mice, which is partially prevented in *arg-ii^-/-^* animals, particularly in males. The improved phenotype of *arg-ii^-/-^* mice in aging is associated with reduced sarcopenia, cellular senescence, inflammation, and fibrosis, whereas age-associated decline of microvascular endothelial cell density, satellite cell numbers, and muscle fiber types in skeletal muscle is prevented in *arg-ii^-/-^* mice. Finally, we demonstrate an increased *arg-ii* gene expression level in aging skeletal muscle and found Arg-II protein expression in endothelial cells and fibroblasts, but not in skeletal muscle fibers, macrophages, and satellite cells. Our results suggest that increased Arg-II in non-skeletal muscle cells promotes age-associated sarcopenia, particularly in male mice.

## INTRODUCTION

Aging is a multifactorial process characterized by the progressive accumulation of tissue damage and degeneration, leading to the functional decline of organs and eventually death of organisms [1, 2]. Among different organs and tissues, skeletal muscle aging is manifested by muscle fiber atrophy and sarcopenia, contributing to frailty and loss of mobility in the elderly [3]. Potential mechanisms of skeletal muscle weakness include cellular senescence, chronic accumulation of inflammatory cells such as macrophages in skeletal muscle, fibrosis, decrease in vascular density and satellite cells (skeletal muscle stem cells), as well as alterations in muscle fiber types, etc., [4–9]. Despite intensive research, the underlying cellular and molecular mechanisms of skeletal muscle aging remain obscure and not fully understood.

In the past, there has been evidence demonstrating that the enzyme arginase plays a significant role in cellular senescence, various organ aging, and lifespan shortening in mouse models [10]. There are two isoenzymes of arginase, i.e., Arg-I and Arg-II [11]. Both Arg-I and Arg-II, encoded by distinct genes, share the same effects on the conversion of L-arginine to L-ornithine and urea [11]. This function of arginase decreases L-arginine bioavailability for the production of the vasoprotective nitric oxide (NO) from endothelial NO-synthase, resulting in endothelial dysfunction and increases the production of polyamines (cell growth factors) and collagen (important for fibrosis) from the metabolite L-ornithine [11]. Arg-I is a cytosolic enzyme which is highly expressed in the liver and essentially involved in the hepatic urea cycle to remove toxic ammonia [12]. Arg-II, however, is found in the mitochondria and expressed abundantly in the kidney [13, 14]. It has been reported that both isoenzymes, particularly Arg-II, are highly upregulated in aging and diseases, which participates in aging and age-related diseases, at least partly due to mitochondrial oxidative stress and exaggerated inflammatory responses and tissue fibrosis in various organs or cells [15–17]. It is interesting to note that the effects of Arg-II are not necessarily dependent on L-arginine metabolism, causing L-arginine depletion and enzymatic activity-independent effects on cell senescence and/or apoptosis have also been reported [18]. Upregulation of Arg-II in aging has been shown in various organs, including heart, blood vessels, pancreas, kidney, and lung [15, 19–22]. Genetic ablation of *arg-ii* or pharmacological inhibition of the arginase enzyme has been shown to reduce cellular senescence, slow down the aging process, improve aging phenotype, and ameliorate age-associated chronic diseases in rodent models [23, 24].

To our knowledge, the role of Arg-II in skeletal muscle aging is not known. Taking into account that skeletal muscle fibers are rich in mitochondria and Arg-II is a mitochondrial enzyme and upregulated with aging [11], this study aims to investigate whether skeletal muscle cells or other cell types in the skeletal muscle such as macrophages, endothelial cells, fibroblasts, and satellite cells express Arg-II, contributing to skeletal muscle aging. in a naturally aging mouse model.

## RESULTS

### *Arg-ii^-/-^* mice reveal improved physical activities in aging

**Figure 1:**
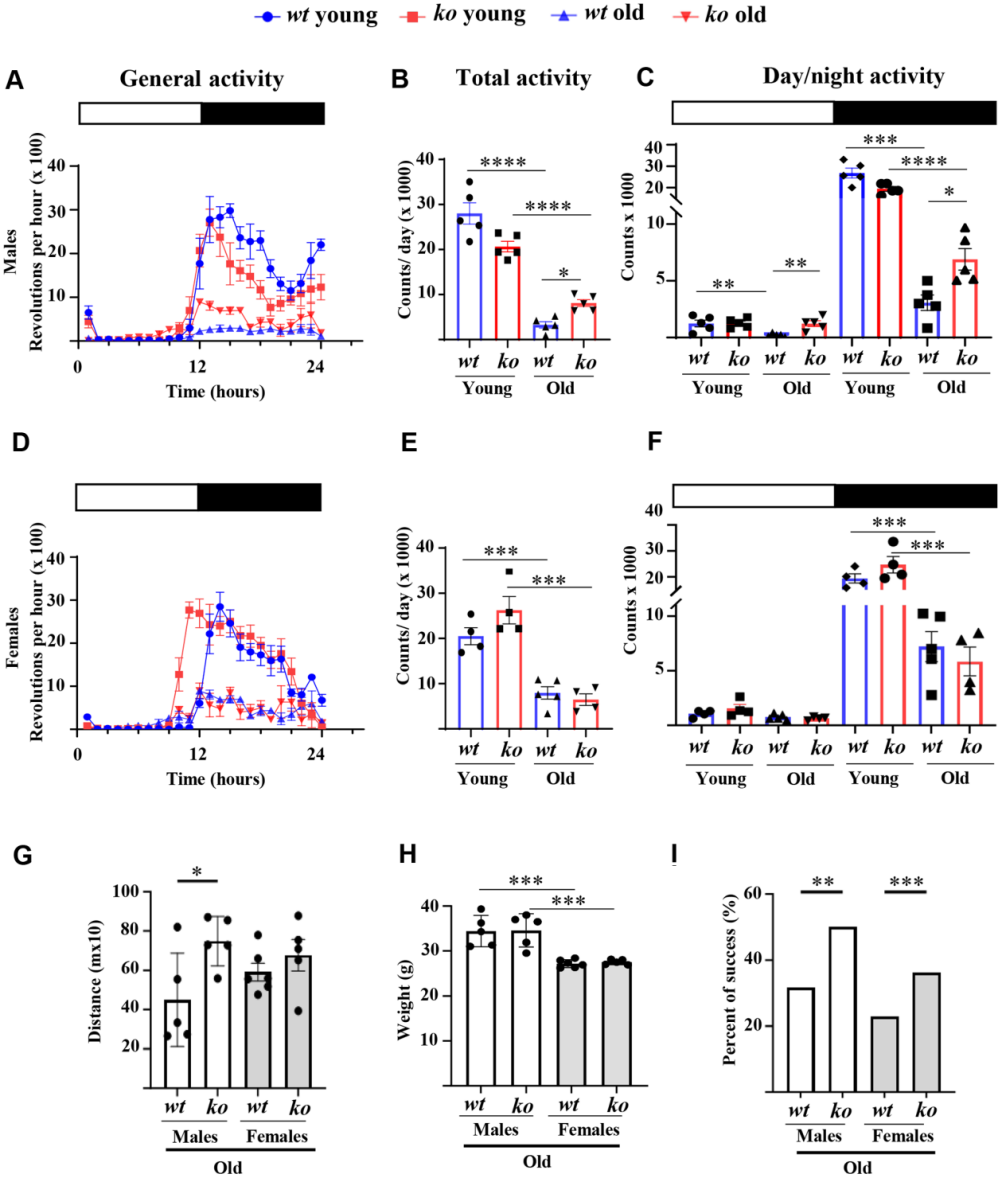
***Arg-ii^-/-^* mice reveal improved physical activity in aging.** Diurnal voluntary physical activities (as revolution/hour on an average of 7 days in the wheel running cages under 12:12 light-dark conditions: 0-12, light-on (resting phase, white bar); 12–24, light-off (active phase, black bar) of young and old wild type (*wt*) and *arg-ii^-/-^* (*ko*) male (**A**–**C**) and female mice (**D**–**F**). (**A**, **D**) General activities of wheel running in males and females respectively; (**B**, **E**) Quantification of the general or total wheel-running activities during light-on and light-off in males and females, respectively. Graphs show the counting of total wheel revolutions during 24 hours in the young and old *wt* and *ko* mice. (**C**, **F**) Graphs show wheel running activities during light-on (white bar) and light-off (black bar) in males and females, respectively. Data are reported as means ± SEM. Combined one-way ANOVA and unpaired *t*-test with Welch’s correction were applied. *n* = 4 to 5 mice for each group. (**G**) Non-voluntary physical activities (treadmill fatigue test) in old male and female *wt* and *arg-ii^-/-^* mice. A parametric unpaired *t*-test with Welch’s correction was applied. *n* = 5 animals for each group. ^*^p < 0.05. (**H**) Body weight in old male and female *wt* and *arg-ii^-/-^* mice. One-way ANOVA was applied with *n* = 5 animals for each group. ^***^p < 0.001. (**I**) Traction test measuring the success rate of lifting their weight to lean all the paws on a horizontally suspended bar. A contingency test was performed with *n* = 11–12 mice for each group. ^*^p < 0.05, ^**^p < 0.01, ^***^p < 0.001, and ^****^p < 0.0001 between the indicated groups.

The daily voluntary physical activities as analyzed by wheel running experiments during 24 hours (Figure 1A) are significantly reduced in old *wt* male mice as compared to the young *wt* male animals (Figure 1A, 1B). The age-associated decline in voluntary activities occurs in both inactive phase (daytime) and active phase (nighttime period) but mainly in the latter phase (Figure 1C). Interestingly, *arg-ii* gene deficiency (*arg-ii^-/-^*) reduced the age-associated decline of voluntary activities during the inactive phase and significantly improved that during the active phase (Figure 1C). Similar to the males, an age-associated decline in voluntary activities was observed in females (Figure 1D). However, no difference in the activities were observed between old *wt* and age-matched *arg-ii^-/-^* females either during the daytime or the nighttime (Figure 1D–1F). Furthermore, a non-voluntary muscle strength test, i.e., treadmill test, demonstrated a significant improvement in running distance in the old *arg-ii^-/-^* male mice as compared to the age-matched *wt* controls (Figure 1G), while this improvement of physical activities was not observed in old female group (Figure 1G). Of note, the body weight in the male group was significantly higher than the female group but was not significantly different within each sex group between *wt* and *arg-ii^-/-^* mice (Figure 1H). The traction test, however, showed an enhanced success rate in the old *arg-ii^-/-^* mice as compared to the aged-matched *wt* animals in both males and females (Figure 1I). Of note, young animals showed a 100% success rate (not shown).

### *Arg-ii^-/-^* mice are partly protected from skeletal muscle aging phenotype

**Figure 2:**
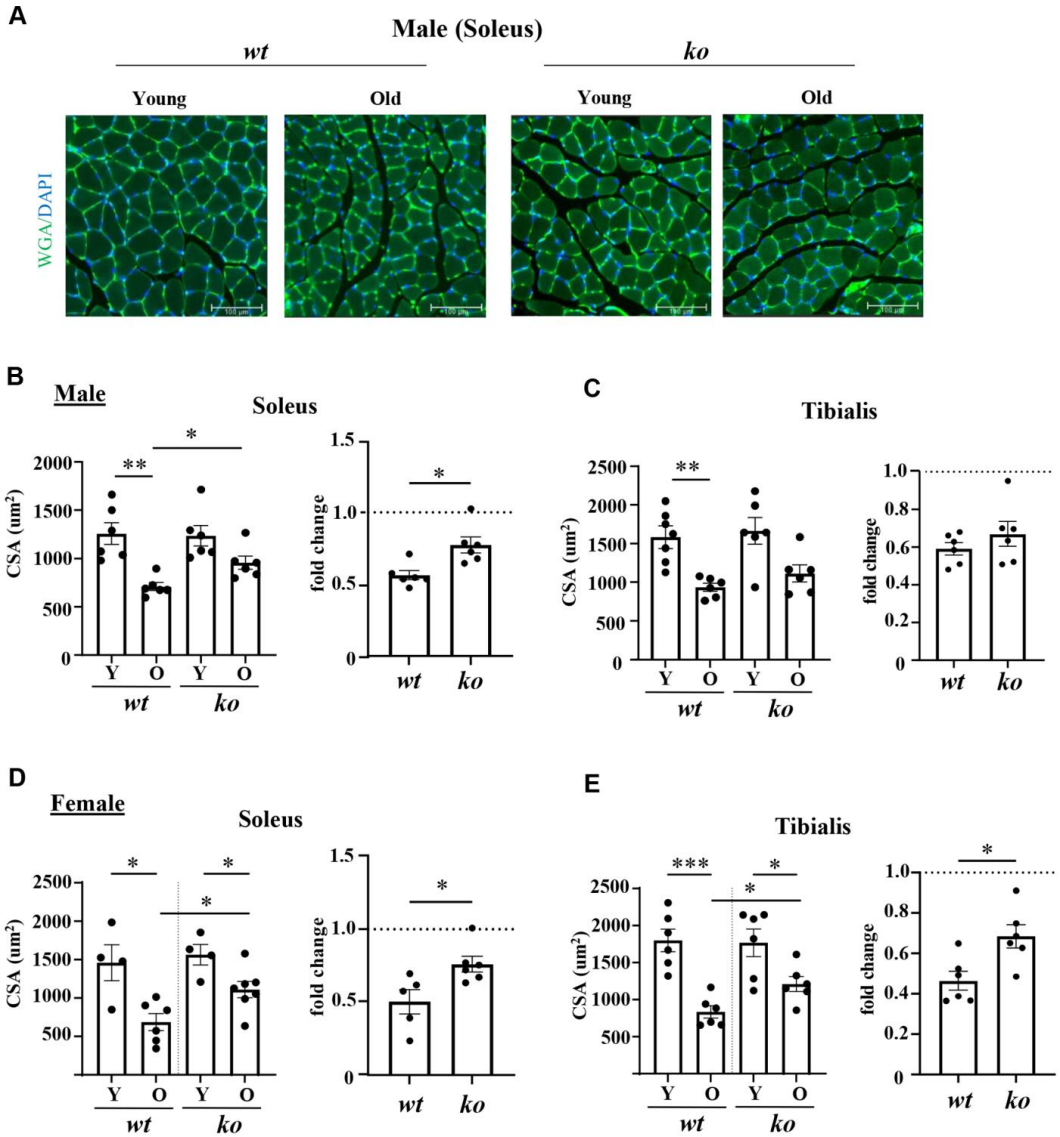
***Arg-ii^-/-^* mice reveal protection against age-associated sarcopenia (muscle fiber cross-sectional area, CSA).** (**A**) Representative images of soleus sections stained for nuclei with DAPI (blue) and skeletal sarcolemma with WGA (green) in young and old wild type (*wt*) and *arg-ii^-/-^* (*ko*) male mice. Scale bar: 100 µm. (**B**–**E**) left panels present quantification of CSA in µm^2^ of soleus and tibialis, in male and female mice, while the right panels present the fold change in CSA based on the ratio of old/young reflecting the age-dependent reduction of CSA. Combined parametric one-way ANOVA and unpaired *t*-test with Welch’s correction were applied; *n* = 4–7 mice for each group. ^*^p < 0.05, ^**^p < 0.01, ^***^p < 0.001 between the indicated groups.

**Figure 3:**
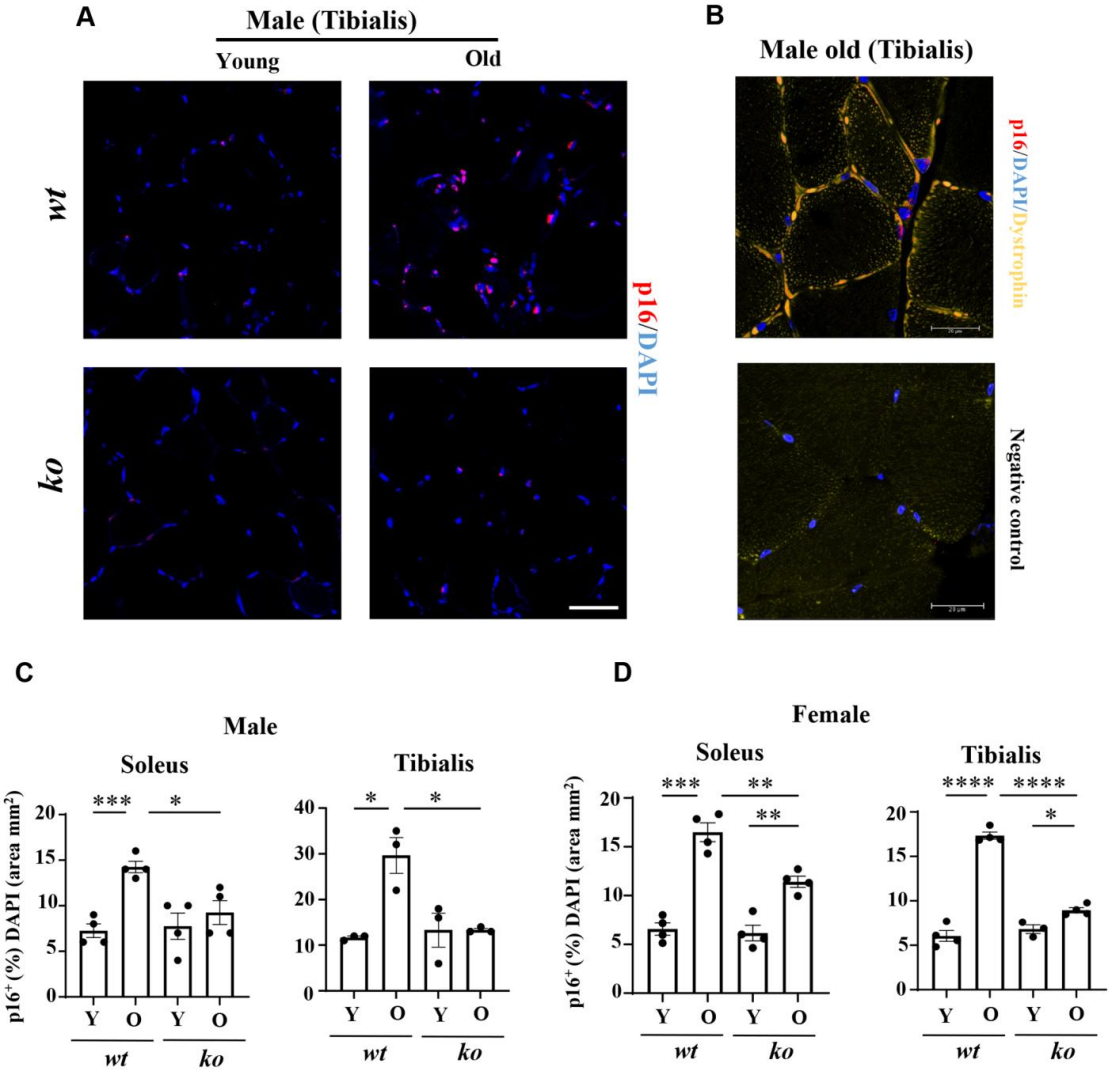
***Arg-ii^-/-^* mice reveal reduced senescent cells in aging skeletal muscles.** (**A**) Representative confocal immunofluorescence staining of DAPI (blue) and anti-p16 (red) in tibialis from young and old wild type (*wt*) and *arg-ii^-/-^* (*ko*) male mice. Scale bar: 50 µm. (**B**) Representative image of old male mouse tibialis stained with DAPI (blue), anti-p16 (red), and anti-dystrophin (yellow, marker of sarcolemma). Negative control staining without the two primary antibodies. Scale bar: 20 µm. (**C**, **D**) Quantification of p16^+^ nuclei (senescent cells) expressed as the percentage of p16^+^ nuclei/total number of nuclei in the mm^2^ area. One-way ANOVA test was applied. *n* = 3–4 mice per group. ^*^p < 0.05, ^**^p < 0.01, ^***^p < 0.001, ^****^p < 0.0001 between the indicated groups.

**Figure 4:**
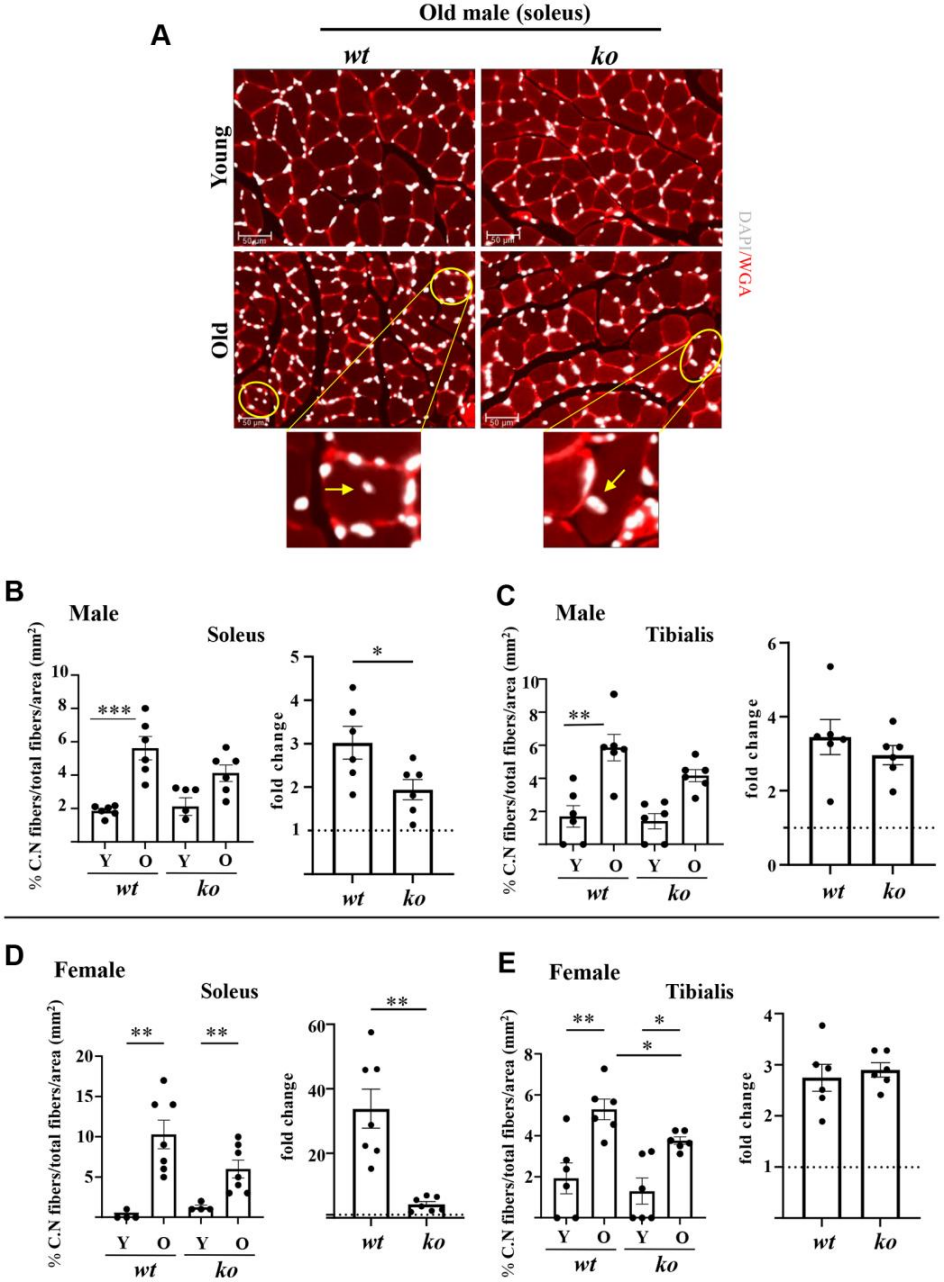
***Arg-ii^-/-^* mice reveal reduced accumulation of centrally located nuclei in aging skeletal muscles.** (**A**) Representative confocal immunofluorescence staining of DAPI (white) and WGA (red) for nuclei and skeletal muscle fiber sarcolemma, respectively, in the old soleus muscle from young and old wild type (*wt*) and *arg-ii^-/-^* (*ko*) male mice. Scale bar: 50 µm. (**B**–**E**) The left graphics in each figure present the percentage of centrally nucleated fibers/total of fibers in the mm^2^ area for both soleus and tibialis in male and female mice as indicated. The fold changes based on the ratio of old/young (dotted line: young as 1) measuring the aging-dependent increase in centrally nucleated fibers were presented in the right bar graph in each panel. Combined one-way ANOVA and unpaired *t*-test with Welch’s correction were applied. *n* = 4–7 mice per group. ^*^p < 0.05, ^**^p < 0.01, ^***^p < 0.001 between the groups.

The soleus and tibialis muscles were isolated and analyzed to study the skeletal muscle aging phenotype. In the *wt* male mice, the myofiber cross-sectional area (CSA) of soleus was significantly decreased with aging (Figure 2A, 2B), which was partly inhibited in the age-matched male *arg-ii^-/-^* mice (Figure 2A, 2B). In the tibialis, however, *arg-ii* deficiency did not significantly affect the age-associated sarcopenia (Figure 2C). The age-associated decrease in CSA was significantly ameliorated in soleus and tibialis of female a*rg-ii^-/-^* mice (Figure 2D, 2E). Moreover, the number of p16^+^ (senescent marker) cells (Figure 3A, 3B) was significantly higher in old male *wt* mice as compared to the young *wt* animals either in soleus or tibialis, which was decreased in age-matched *arg-ii*^-/-^ mice (Figure 3C). Similar findings on the inhibitory effects of *arg-ii*^-/-^ on cell senescence were observed in aged female mouse muscles (Figure 3D). The p16^+^ nuclei were confirmed to be the skeletal muscle fibers (e.g., in tibialis of the male mice) by performing co-immunostaining of p16 and dystrophin (Figure 3B). Furthermore, a significant age-associated increase in the number of centralized nuclei in soleus of male mice was observed, which was reduced in age-matched *arg-ii^-/-^* animals (Figure 4A, 4B). A similar trend of *arg-ii^-/-^* inhibitory effect on age-associated increase in centralized nuclei was observed in tibialis. This, however, did not reach statistical significance (Figure 4C). The age-associated increase in centralized nuclei was also significantly decreased in *arg-ii^-/-^* female mouse soleus but not in tibialis (Figure 4D, 4E).

### *Arg-ii* deficiency reduces inflammation and improves microvascular density in aging skeletal muscle

**Figure 5:**
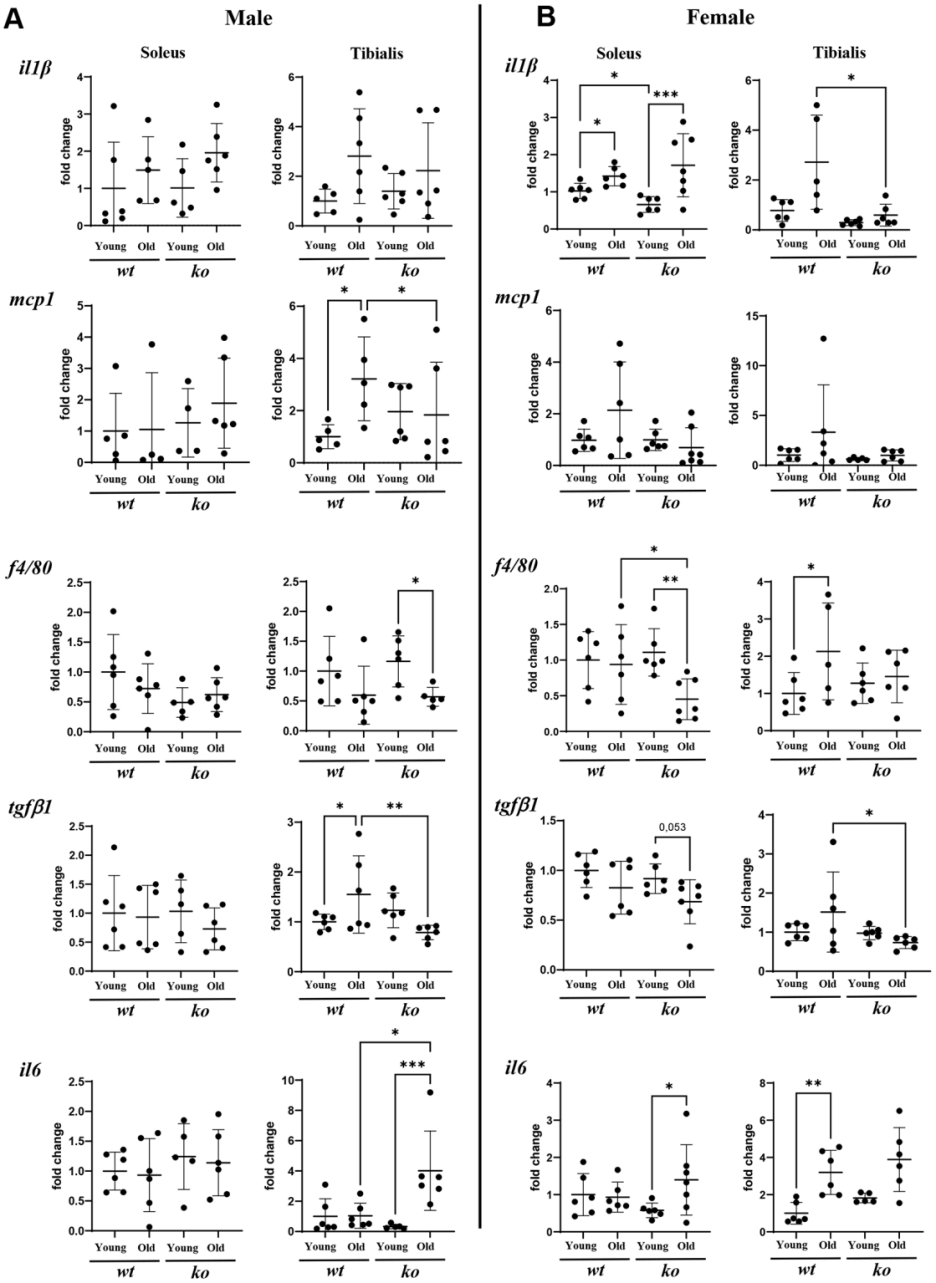
***Arg-ii^-/-^* mice reveal decreased inflammatory markers in aging skeletal muscles.** mRNA expression levels of inflammatory markers and cytokines analyzed by qRT-PCR in soleus and tibialis from male (**A**) and female (**B**) young and old wild type (*wt*) and *arg-ii^-/-^* (*ko*) mice. One-way ANOVA was applied. *n* = 4–7 mice per group. ^*^p < 0.05, ^**^p < 0.01, ^***^p < 0.001 between the groups.

**Figure 6:**
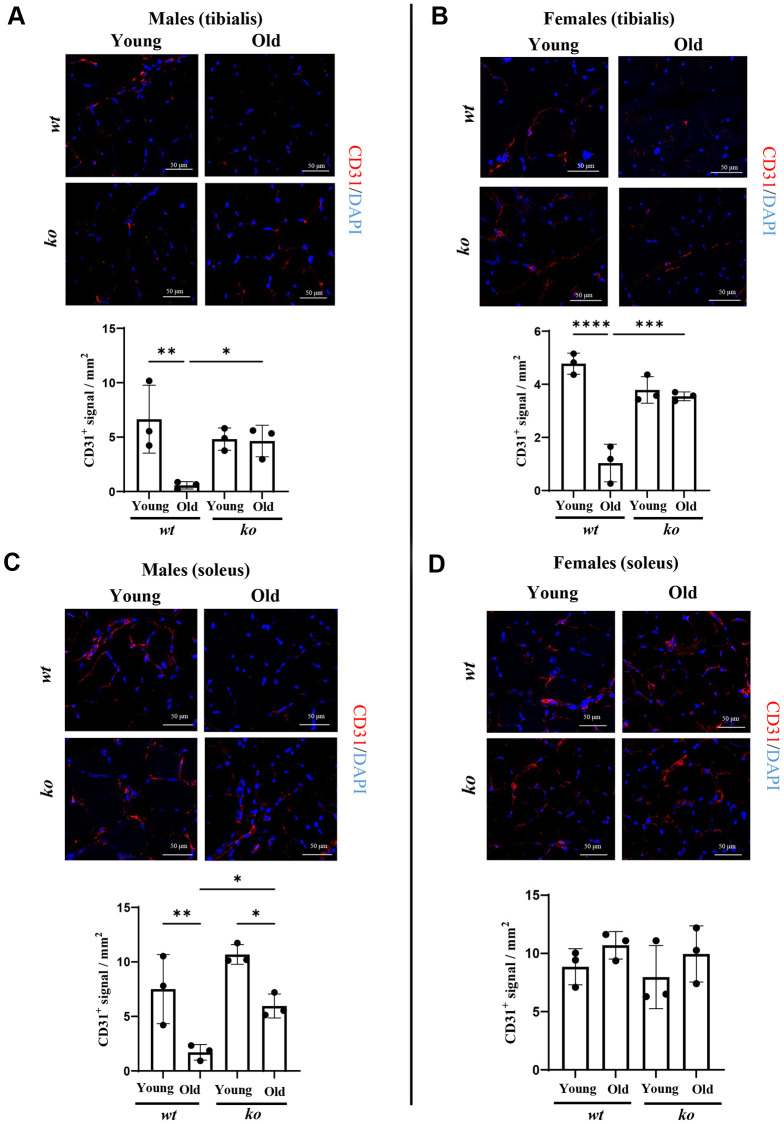
***Arg-ii^-/-^* mice reveal preserved microvascular density in aging skeletal muscle.** (**A**) Representative confocal images showing CD31 staining (red; endothelial cells marker) in male tibialis of young and old male wild type (*wt*) and *arg-ii^-/-^* (*ko*) mice. DAPI (blue) is used to stain the nuclei. The graph reports the quantification of CD31 signal in the area (mm^2^). Scale bar: 50 µm. (B to D) Representative confocal images and relative quantification for CD31 (red) and DAPI (blue) in tibialis from young and old female *wt* and *ko* mice (**B**), and in soleus of young and old male (**C**) and female (**D**) *wt* and *ko* mice. Scale bar: 50 µm. One-way ANOVA was applied with *n* = 3 mice per group. ^*^p < 0.05, ^**^p < 0.01, ^***^p < 0.001, ^****^p < 0.0001 between the groups.

Since age is associated with chronic inflammation, the role of *arg-ii* in skeletal muscle inflammation was analyzed in soleus and tibialis of old *wt* and *arg-ii^-/-^* mice. Gene analysis of several inflammatory cytokines in the skeletal muscle tissues from male mice revealed that there was a significant reduction of *mcp1* (male) and *tgfb1* in the tibialis (not soleus) in *arg-ii^-/-^* as compared to age-matched *wt* animals (Figure 5A). No consistent inhibitory effect of *arg-ii^-/-^* on other inflammatory markers such as *Il-1b*,* f4/80, Il6* was observed (Figure 5A). In the female mice, *arg-ii^-/-^* reduced *Il-1b*, *mcp1, f4/80, tgfb1* in either one or two skeletal muscles (Figure 5B). Immunofluorescence confocal staining confirmed the age-associated increase in F4/80^+^ cells (macrophages) in tibialis of male mice, which was reduced in age-matched *arg-ii^-/-^* animals (Supplementary Figure 1). Importantly, an age-associated decrease in CD31^+^ cells (an endothelial marker) was observed in tibialis and soleus of *wt* male mice, which was prevented in age-matched *arg-ii^-/-^* animals (Figure 6A, 6C). This preventive effect of *arg-ii^-/-^* was also observed in the female tibialis muscle (Figure 6B). However, no age-associated decrease in CD31^+^ signals and no significant effects of *arg-ii^-/-^* were observed in the female soleus (Figure 6D).

### *Arg-ii* deficiency reduces fibrosis in aging skeletal muscle

**Figure 7:**
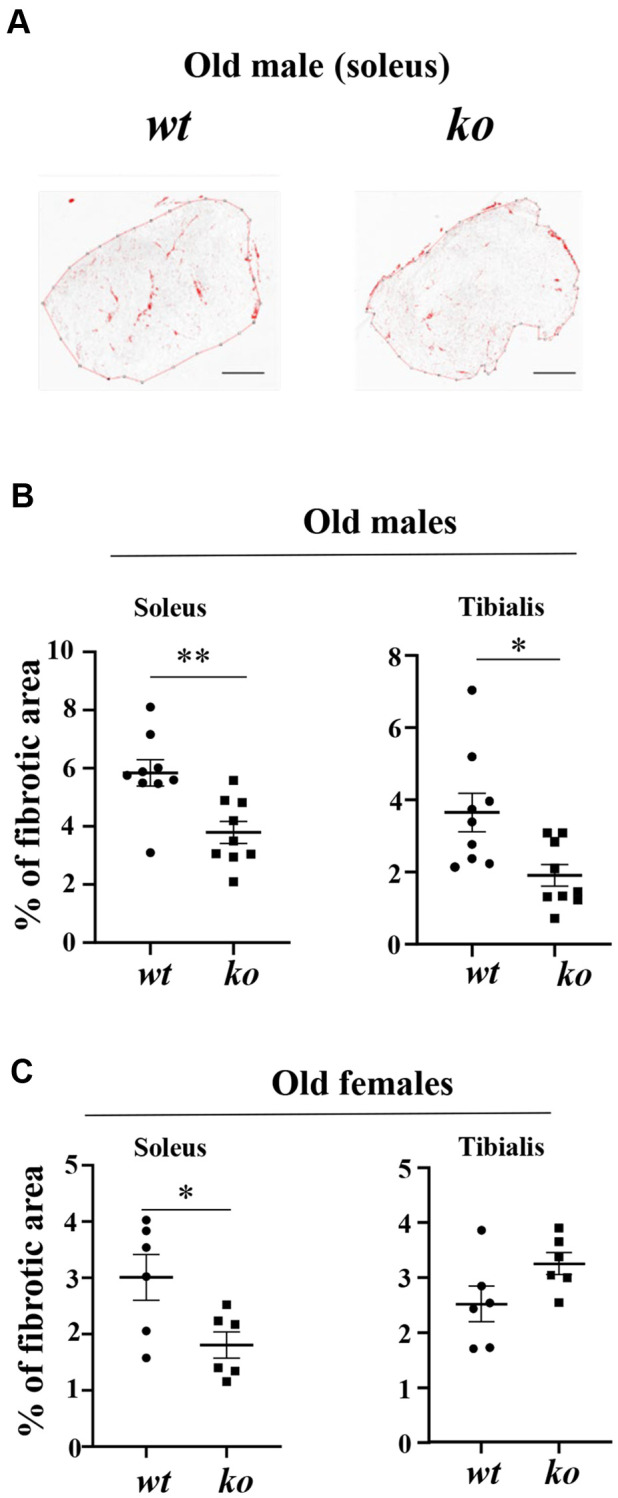
***Arg-ii^-/-^* mice reveal reduced skeletal muscle fibrosis in aging.** (**A**) Representative masking images showing collagen signals (red) in soleus from old male wild type (*wt*) and *arg-ii^-/-^* (*ko*) mice. (**B**, **C**) Quantification of fibrosis area in both soleus and tibialis from old males and females. A parametric unpaired *t*-test with Welch’s correction was applied. *n* = 6–9 mice per group. ^*^p < 0.05, ^**^p < 0.01 between the groups. Scale bar: 250 µm.

Age-associated skeletal muscle fibrosis, although mild, was observed in the soleus and tibialis of male *wt* mice, which was reduced in age-matched *arg-ii^-/-^* animals (Figure 7A, 7B). Similar results on inhibition of skeletal muscle fibrosis in *arg-ii^-/-^* mice were demonstrated in the female mouse soleus but not in the tibialis (Figure 7C), demonstrating partial prevention of age-associated muscle fibrosis by *arg-ii* deficiency. No effects of *arg-ii* deficiency on the expression of *col1α1* or *col3α1* assessed by qRT-PCR were observed in males or females of old mice (Supplementary Figure 2A, 2B).

### *Arg-ii* deficiency prevents age-associated decrease in type-I (slow) and type-IIa (fast) muscle fibers

**Figure 8:**
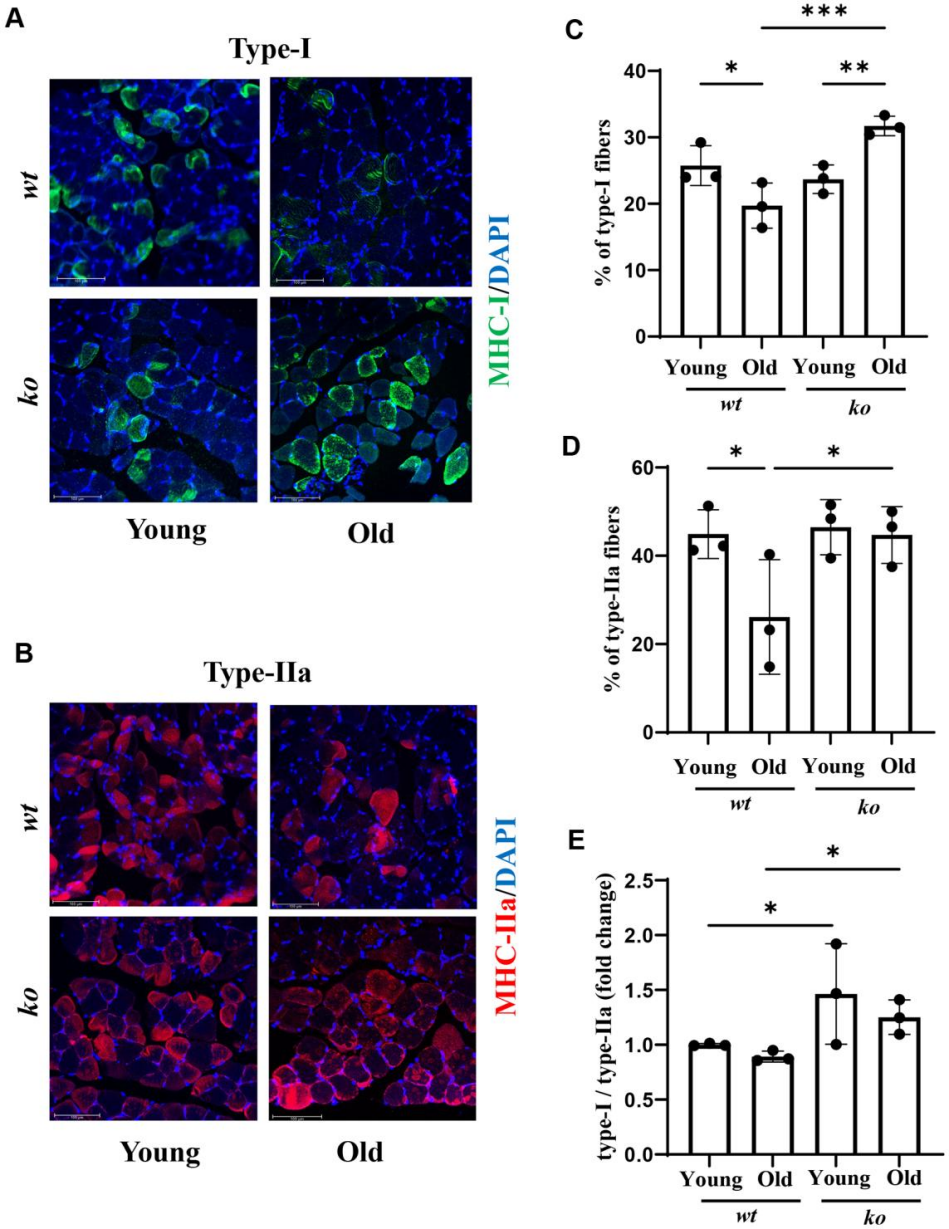
***Arg-ii* deficiency prevents age-associated decrease in type-I and type-II muscle fibers.** Representative confocal images showing Type-I (**A**) and Type-IIA (**B**) fibers in the soleus of young and old *wt* and *arg-ii^-/-^* (*ko*) male mice. DAPI (blue) is used to stain nuclei. Scale bar = 100 µm. The graphs present the percentage of Type-I (**C**) and Type-II (**D**) fibers over the total number of fibers in the image. (**E**) the graph shows the ratio of Type-I and Type-II fibers expressed as fold change (*wt* young group was taken as reference). One-way ANOVA test was applied. *n* = 3 mice per group. ^*^p < 0.05, ^**^p < 0.01, ^***^p < 0.001 between the indicated groups.

Since the tibialis muscle in mice does not have much type-I and type-IIa [25], which is in contrast to the soleus muscle (Supplementary Figure 3), the muscle fiber types were therefore analyzed in the soleus. As shown in Figure 8A–8D, there was an age-associated decrease in both type-I and type-IIa fibers in the soleus muscle of male mice, which was prevented in age-matched *arg-ii^-/-^* animals. A similar finding was observed in female soleus for type-I (Supplementary Figure 4A, 4C), but not type-IIa fibers (Supplementary Figure 4B, 4D). Of note, the portion of type IIa fibers in female soleus was very low (Supplementary Figure 4B) and the type-I/type-IIa ratio was much higher in females than males of both *wt* and *arg-ii^-/-^* animals (Supplementary Figure 5). Interestingly, the ratio of muscle fiber type-I/type-IIa was not significantly affected by aging (Figure 8E). However, *arg-ii^-/-^* significantly enhanced type-I/Type-IIa ratio in males (Figure 8E), but not in females (Supplementary Figures 4E and 5).

### Increased *arg-ii* levels and cellular localization of Arg-II in aging skeletal muscle

**Figure 9:**
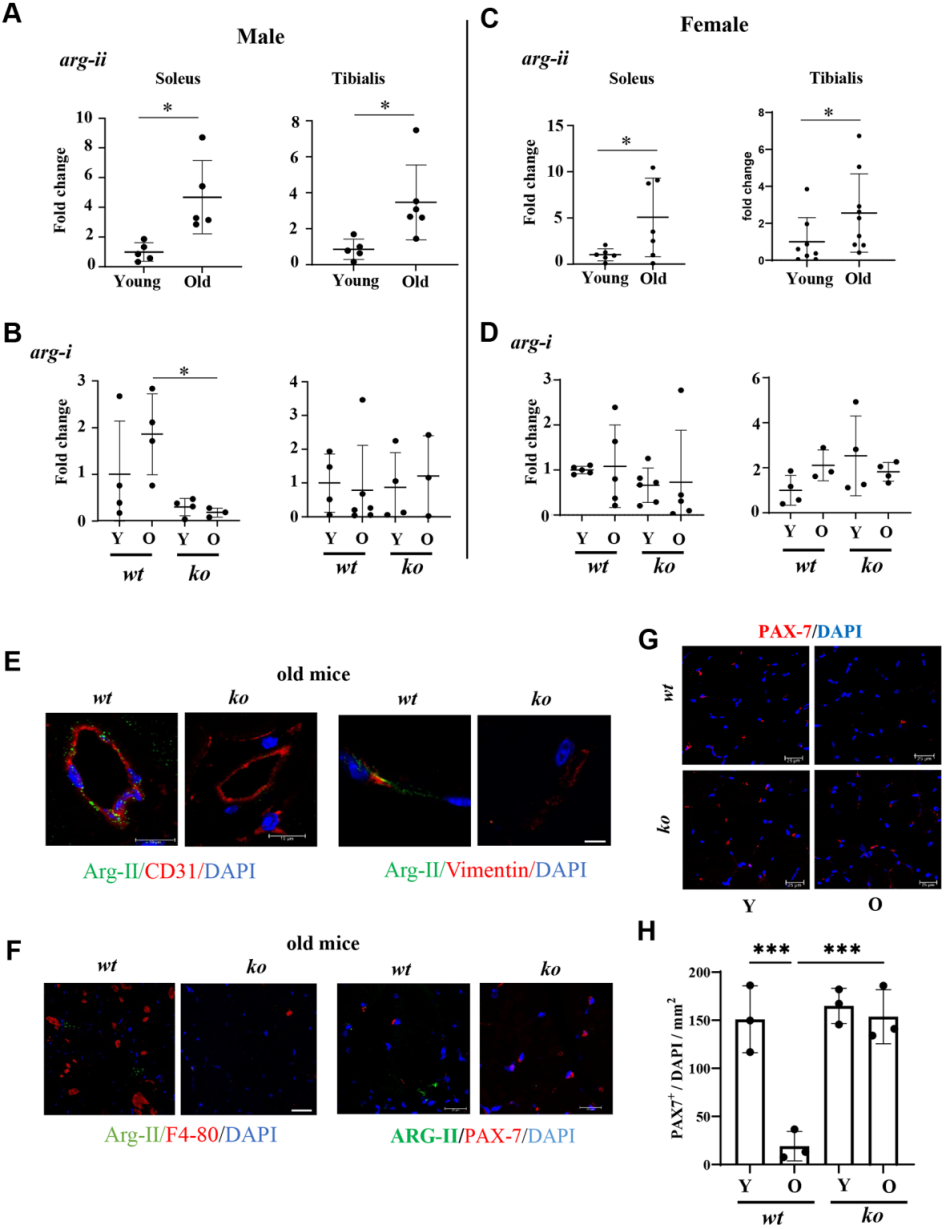
***Arg-ii* expression and cellular localization in aging skeletal muscles.*** Arg-ii* mRNA levels were analyzed by qRT-PCR in skeletal muscles of wild-type (*wt*) male (**A**) and female (**C**) mice. (**B**, **D**) *arg-i* expression in soleus and tibialis from young and old *wt* and *ko* mice in both sexes. A parametric unpaired *t-*test with Welch’s correction was applied. *n* = 3–9 mice per group. ^*^p < 0.05 between the indicated groups. (**E**) Representative confocal images of old *wt* and *ko* skeletal muscle showing co-localization of Arg-II (green) with CD31 (red, endothelial marker), and with Vimentin (red, fibroblasts marker). (**F**) Representative confocal images of old *wt* and *ko* muscle showing lack of co-localization of Arg-II (green) with F4-80 (red, macrophage marker), and with PAX-7 (red, satellite cells marker). DAPI (blue) stains cell nuclei. Scale bar: 10 µm (Arg-II/CD31); 25 µm (Arg-II/Vimentin); 10 µm (Arg-II/F4/80); 20 µm (Arg-II/PAX-7). Each experiment was repeated with 3 animals. (**G**) Representative confocal images of PAX-7 (red) in skeletal muscle of young and old *wt* and *arg-ii^-/-^* mice. Scale bar: 25 µm. (**H**) Quantification of PAX-7^+^ cells (satellite cells) in skeletal muscle expressed as the percentage of PAX-7^+^/total number of cells in the mm^2^ area. One-way ANOVA test was applied. *n* = 3 mice per group. ^***^p < 0.001 between the indicated groups.

In soleus and tibialis of both males and females, *arg-ii* expression levels detected by qRT-PCR are significantly increased in aging (Figure 9A, 9C), while no significant changes in *arg-i* were observed with aging (Figure 9B, 9D) and no significant effects of *arg-ii^-/-^* on *arg-i* expression were found in male and female animals, except a decrease in *arg-i* expression in the old *arg-ii*^-/-^ male soleus as compared to age-matched *wt* animals (Figure 9B). However, immunoblotting was unable to demonstrate Arg-II protein signals (data not shown), suggesting low expression levels of Arg-II in the skeletal muscle. Therefore, confocal immunofluorescence staining was performed for cellular localization of Arg-II in the skeletal muscle of aged animals. Arg-II was not found in skeletal muscle fibers, which was, to our surprise. Arg-II was however found in CD31^+^ endothelial cells and vimentin^+^ fibroblasts (Figure 9E). Arg-II was not found in the F4/80^+^ macrophages in the skeletal muscle of aged mice (Figure 9F). It is also not found in Pax7^+^ satellite cells (Figure 9F). Interestingly, Pax7^+^ satellite cells were decreased in aging *wt* mice, which was prevented in *arg-ii*^-/-^ mice (Figure 9G, 9H). Co-immunostaining demonstrated that vimentin^+^ fibroblasts and F4/80^+^ macrophages expressed TGFβ-1 (Supplementary Figure 2C), indicating a role of Arg-II in the activation of fibroblasts.

## DISCUSSION

Our findings of this study show that *arg-ii* gene expression is increased in skeletal muscle in a naturally aging mouse model and *arg-ii* gene knockout reduces age-related sarcopenia, inflammation, cell senescence, and skeletal muscle tissue fibrosis, prevented age-associated decrease in myofiber types including type-I and type-II fibers, particularly in males. Moreover, age-associated decline of satellite cells (muscle stem cells) is prevented in *arg-ii^-/-^* animals. These beneficial effects of *arg-ii* gene knockout on skeletal muscle aging are correlated with the improvement of muscle strength and physical activities.

As reported in the literature [26], our study shows an age-related decline in locomotor activity in the naturally aging *wt* mice as assessed by the wheel-running test. The decreased physical activities in aged mice occur during the daytime (inactive phase) and mainly during the nighttime (active phase) as expected. Interestingly, mice lacking *arg-ii* (*arg-ii^-/-^*) show a higher locomotor activity as compared to the *wt* age-matched animals. Surprisingly, the improved locomotor activity in aged *arg-ii^-/-^* mice is observed only in males but not in females. Similarly, the endurance of physical activity as analyzed by treadmill test is significantly improved in old *arg-ii^-/-^* males but not the *arg-ii^-/-^* females. However, the traction test, which reflects a combination of muscle strength and movement coordination suggests activity improvement in both males and females in aging. This finding suggests that *arg-ii^-/-^* in aging improves physical activities through maintaining muscle health and probably also neuro-muscle coordination function in both sexes. This aspect requires further investigation. Of note, there is no difference in body weight between *wt* and *arg-ii^-/-^* mice of either males or females, excluding an influence of body weight on physical activities within the sex group. Overall, the results indicate a role of *arg-ii* in the age-associated decline of physical activities in both sexes. Notably, these improving effects of *arg-ii* deficiency on physical activities are more pronounced in males than in females. The underlying mechanisms of the sex-biased effects of *arg-ii* deficiency remain obscure. It is interesting to note that a significantly enhanced ratio of type-I/type-IIa muscle fibers is observed in the male but not female *arg-ii^-/-^* mice, although other subtypes of type-II muscle fibers are not analyzed in this study. Moreover, female mice have a much higher portion of type-I fibers than male animals, which is similar to humans [27]. Considering that type-I fibers are slow twitch, use aerobic respiration to produce ATP, and are relatively resistant to fatigue [28, 29], and the above-mentioned differential effects of *arg-ii* deficiency on muscle fiber distribution in males and females, this may at least partly explain the more pronounced effect of *arg-ii* gene deficiency on improved physical activity in the males than in females with aging. It is to be noted that both type-I and type-IIa fibers are reduced with age at least in the soleus of both males and females, which is prevented by *arg-ii* deficiency. How *arg-ii* exactly regulates fiber types in males and females remains an interesting topic for further investigation. The observation is in line with the previous study showing the beneficial effects of *arg-ii* deficiency on age-associated renal podocyte injury and albuminuria in male mice but not in female mice [30]. Whether the observed sex-related difference in the phenotype is related to sex hormones remains to be investigated. Taking into account that Arg-II is expressed in the brain [31] and an improved voluntary physical activity (wheel running) during the night was observed in old males but not in age-matched females, a different effect of *arg-ii* deficiency on neuronal activities controlling neuromuscular function between males and females may exist. This aspect warrants further investigation.

The age-associated reduced locomotion, strength, and endurance are associated with skeletal muscle atrophy as demonstrated by the decreased cross-sectional area (CSA) of the muscle fibers in the soleus or tibialis in males and females. Interestingly, this age-associated sarcopenia is in general partly prevented in the *arg-II^-/-^* mice, although this prevention is not always observed in all the muscles studied. The CSA reduction associated with aging can result from a multifactorial process, including the accumulation of senescent cells [4], decreased vascular density [7], chronic inflammation [6], and muscle tissue fibrosis [32]. Indeed, we found that *arg-ii* deficiency reduced several characteristics of aging skeletal muscle phenotype in the soleus or tibialis, including senescent skeletal muscle cells, inflammatory markers such as *mcp1, il-1β, tgfb1* and *f4/80,* although there are some differences between male and female mice and between tibialis and soleus. It is surprising to observe that among these cytokines, the expression levels of *Il6* are significantly higher in *arg-ii^-/-^* mice as compared to the *wt* control mice with aging. This may implicate muscle reactive regeneration capacity in response to injury since *Il6* expression is associated with skeletal muscle function and regeneration [33]. Moreover, the fact that the number of centrally nucleated myofibers, an indicator of the degeneration and regeneration of muscle fiber [34], is increased in aged* wt* mice, which is reduced in age-matched *arg-ii^-/-^* animals, further suggests a protective effect on of skeletal muscle degeneration by *arg-ii* deficiency. It is also interesting to notice that an age-related decrease in satellite cells in muscle fibers is prevented in *arg-II^-/-^* mice. Finally, the beneficial effects of *arg-ii^-/-^* are also reflected by an improved capillary endothelial density in muscle fibers in the age-matched *arg-ii^-/-^* mice as compared to the *wt* counterparts, although in females the significant effects of aging and *arg-ii* gene knockout on capillary density could be only observed in the tibialis but not soleus. This sex-related difference may also partly contribute to the more pronounced effect of *arg-ii* deficiency on physical activities in old male animals.

The question is how *arg-ii* deficiency protects against age-associated skeletal muscle degeneration. To our surprise, the mitochondrial enzyme Arg-II is not found in the skeletal muscle fibers which are rich in mitochondria. This finding is similar to that in cardiomyocytes which are also rich in mitochondria but do not express Arg-II [35]. The deteriorative effect of Arg-II in aging muscle fibers must be due to the non-cell-autonomous function of the molecule, i.e., Arg-II might mediate the aging-dependent muscle degeneration and sarcopenia in a paracrine manner, a phenomenon demonstrated in the aging lung [15] and aging heart [35]. Indeed, Arg-II is found in fibroblasts and vascular endothelial cells, but not in macrophages and satellite cells. It has been shown that Arg-II level is upregulated in aging endothelial cells and plays a crucial role in endothelial dysfunction, i.e., decreased endothelial NO bioavailability [9]. Endothelial NO is able to stimulate angiogenesis [36]. It is presumable that increased endothelial NO levels in aged *arg-ii^-/-^* as shown previously [19] improves endothelial angiogenesis [36]. This may explain the preservation of capillary endothelial density in skeletal muscle fibers in aged *arg-ii^-/-^* mice. Moreover, Arg-II is expressed in fibroblasts in the old skeletal muscle and is capable of activating fibroblasts to produce and secrete inflammatory cytokines and collagen, contributing to tissue degeneration and fibrosis [15, 35]. These cell autonomous effects of Arg-II could at least partly explain the skeletal muscle aging phenotype, i.e., decreased endothelial density and interstitial fibrosis, which is significantly inhibited in *arg-ii^-/-^* mice. In the aging lung and aging heart, fibroblasts express Arg-II, which promotes TGF-β1 production and contributes to enhanced matric production and organ fibrosis associated with aging [15, 35]. Similarly, in the present study, we show that Arg-II is found in fibroblasts and TGF-β1 was expressed in these cells in aging skeletal muscles. This result further suggests that Arg-II and TGF-β1 play a role in age-associated skeletal muscle fibrosis. Indeed, an increased level of *tgf-β1* and skeletal muscle fibrosis in aging is prevented in a*rg-ii^-/-^* mice. Future experiments shall analyze the function and mechanisms of Arg-II in skeletal muscle fibroblasts in age-associated sarcopenia.

It has been shown that Arg-II, in contrast, to Arg-I, promotes macrophage inflammatory responses, contributing to the development of tissue inflammation, i.e., in obesity-associated insulin resistance, glucose intolerance, as well as high cholesterol-diet-induced atherosclerosis in mouse models [37]. Recently, we also confirmed the function of Arg-II in macrophages in the aging heart [35]. To our surprise, Arg-II is not found in the macrophages in aging skeletal muscle in our present study. The enhanced macrophage markers must be due to a paracrine mechanism such as an inflammatory microenvironment contributed by senescence cells which results in senescence-associated secretory phenotype (SASP) [38]. It seems that in humans and also in BALB/c mice, anti-inflammatory M2 macrophages are predominant in aging skeletal muscle [39], contributing to age-associated skeletal muscle fibrosis as shown in mice [40]. Therefore, the inhibition of age-associated skeletal muscle fibrosis could be at least partly explained by decreased expression levels of several inflammatory cytokines and *f4/80* macrophage markers in the old *arg-ii^-/-^* mice, which mirrors the reduced number of senescent cells observed in the a*rg-ii^-/-^ mice* compared to *wt* control animals. Studies demonstrate that senescent cells are metabolically highly active cells secreting growth factors, cytokines/chemoattractants, and proteases, a robust SASP, and are contributing to the inflammatory microenvironment, inflammatory cell infiltration, tissue damage, and fibrosis [41, 42]. In the present study, we observed inhibition of age-associated increase in senescent cells of skeletal muscle fibers in *arg-ii^-/-^* mice. This finding is in line with our previous research work showing an important role of *arg-ii* in cell senescence and organ aging [15, 19, 22].

One of the interesting findings of our study is that the age-associated decline of satellite cells is prevented by *arg-ii^-/-^*. However, we could not observe Arg-II expression in these cells, indicating non-cell autonomous effects of Arg-II on satellite cells in aging. The decreased satellite cell number and functional decline of the cells during aging are complex and regulated by intrinsic and extrinsic cues which are largely derived from numerous communicative stromal cell types in their niche of muscle fibers [9]. It is presumable that other cell types such as endothelial cells and fibroblasts, perhaps other stromal cell types which express Arg-II and become senescent, exhibit SASP, and produce inflammatory cytokines, ROS, etc., to interact with satellite cells, contributing to satellite cell exhaustion and/or functional decline. This aspect remains an interesting topic for future investigation.

In conclusion, Arg-II is not expressed in skeletal muscle fibers but expressed in fibroblasts and microvascular endothelial cells in aging skeletal muscles, which contributes to age-associated inflammation, fibrosis, cell senescence, decreased vascular density, alterations of muscle fiber types, and satellite cell exhaustion, resulting in sarcopenia. This aging skeletal muscle phenotype is reduced in a*rg-ii^-/-^* mice, representing a novel mechanism of age-associated skeletal muscle weakness. Targeting Arg-II could be a novel strategy for improving skeletal muscle functional decline in aging. Since Arg-II was found in vascular endothelial cells and fibroblasts in skeletal muscles of aged mice, which indicates the roles of these cell types in skeletal muscle aging phenotype development through a non-cell autonomous effect or a paracrine effect, future experiments shall investigate the detailed cellular and molecular mechanisms, how Arg-II in these cell types accelerates skeletal muscle fiber degeneration during the aging process. Another interesting observation in this study is the activity phase shift to an earlier time in the young *arg-ii^-/-^* animals of females. This earlier anticipation of the onset may be related to the role of Arg-II in the regulation of the central circadian clock in the brain. Since Arg-II is expressed in the brain [43], it is presumable that Arg-II might be involved in the regulation of circadian rhythms of animals. This aspect requires further exploration in the future.

## MATERIALS AND METHODS

### Materials and reagents

Reagents were purchased from the following sources: Trichrome Stain Kit (ab150686) from Abcam^™^; Wheat Germ Agglutinin (WGA) – Alexa Fluor^®^ 488 Conjugate (W11261), 4′,6-diamidino-2-phenylindole, dihydrochloride (DAPI) (D1306), goat anti-rabbit IgG (H + L) secondary antibody Alexa Fluor^®^ 488 Conjugate (A-1108), goat anti-mouse IgG (H + L) secondary antibody Alexa Fluor^®^ 488 Conjugate (A11001), and goat anti-mouse IgG (H + L) secondary antibody Alexa Fluor^®^ 633 Conjugate (A21050) were purchased from Invitrogen^™^.

### Animals

A*rg-ii* knockout (*arg-ii*^-/-^) mice were kindly provided by Dr. William O’Brien [44] and backcrossed to C57BL/6J for more than 10 generations. Mice were housed in standard mouse cages at 23°C with light: dark (L:D) cycles of 12:12 hours. Animals were fed a normal chow diet and had free access to water and food (ad libidum). Offspring of *wild-type* (*wt*) and *arg-ii^−/−^* mice were generated by interbred from hetero/hetero cross. Genotypes of mice were confirmed by polymerase chain reaction (PCR) as previously described [45]. Healthy male and female mice at the age of 4–6 months (young) or 20–24 months (old), based on a score sheet were included in the study. All experimenters were aware of the group allocation at the different stages of the experiments. At the end of the experiments, animals were euthanized under deep anesthesia (i.p. injection of a mixture of ketamine/xylazine 50 mg/kg and 5 mg/kg, respectively), and death was confirmed by absence of all the reflexes and by exsanguination. The tibialis anterior and soleus were isolated and snap-frozen in liquid nitrogen and kept at −80°C until use. Parts of the tibialis and soleus muscles were cut transversely, fixed with 3.7% paraformaldehyde, and then embedded in paraffin for immunofluorescence staining experiments. Experimental work with animals was approved by the Ethical Committee of the Veterinary Office of Fribourg Switzerland (2021-20-FR) and was performed in compliance with guidelines on animal experimentation at our institution.

### Wheel-running experiment assessing general locomotor activities

Mice were kept under a 12:12 light: dark cycle for ten days in wheel-running cages. Locomotor activity parameters (general activity measured as revolution/hour) of both *wt* and* arg-ii^-/-^* mice were analyzed by monitoring wheel-running activity as previously described [46]. Total daytime and nighttime activities and activity distribution profiles were calculated using the respective inbuilt functions of the ClockLab software (Acquisition Version 3.208, Analysis Version 6.0.36).

### Traction test assessing muscle strength

The traction test was performed as an adapted version of a published protocol [47] to determine the muscle strength of the mice. The platform comprises a 0.2 cm-diameter horizontal wire of 70 cm long, placed at 40 cm height. Before training and testing mice, the traction test wire was ensured on a flat surface. The forepaws of the mice were placed in the center of the metallic wire. Four consecutive attempts were recorded for five consecutive days (after two days of adaptation) to evaluate the performance. The capability of mice to lift their hind paws to the bar and grab it for at least 5 seconds was considered a successful performance. The percentage of the performance success rate was calculated and used as an indicator of muscle strength. Mice who fell or could not complete the task within one minute were considered invalid and excluded from the analysis.

### Treadmill fatigue test assessing endurance activities

The treadmill test defines fatigue-like behavior. The experiments were performed using Ugo Basile^®^ Rodent Treadmill NG (Catalog No. 47300). Mice were initially adapted to physical exercise by challenging them on the running wheel for five days (voluntary activity). Subsequently, mice were adapted to the treadmill for five following days starting with a speed of 3 meters per minute and ramping up to 17 meters per minute at day 5. A dark spot towards the end of the ramp was created and mice were encouraged to walk to the end of the tapis. The treadmill experimental test was performed on a speed starting from 3 meters per minute ramping up to 24 meters per minute in the same trial. When animals stay on the metal grid at the starting point of the ramp, they receive a little electric stimulus to motivate them to run. At the beginning of each session, treadmill inclination at 0% was verified, and all mice were weighted. Treadmill training was performed at the same time of day for five days to exclude confounding variables such as the diurnal oscillation of animal activity. The criteria of termination for this specific experiment were the mouse getting 36 electric stimuli. After the last electric stimulus, we considered that mice reached fatigue. The duration and distance run by each animal before getting 36 stimuli were measured with the software X-PAD3 (Ugo Basile^®^, Italy).

### Cross-sectional area and centrally nucleated fibers analysis

Tibialis anterior and soleus muscles from *wt* and *arg-ii^-/-^* mice were isolated and fixed with 3.7% paraformaldehyde and embedded in paraffin. Sections were cut into 5 μm with a Microm Cool-Cut machine (HM325, Thermo Fisher Scientific, Waltham, MA, USA). After deparaffinization in xylene and hydration in ethanol, antigen retrieval was performed in a pressure cooker to unmask tissue antigens. Transverse sections were stained with Wheat Germ Agglutinin (WGA, 10 μg/ml for 30 minutes) – Alexa Fluor^®^ 488 Conjugate for skeletal fiber sarcolemma to determine cross-sectional area, followed by counterstaining with DAPI. Immunofluorescence signals were visualized under Leica DM6B Navigator and analyzed using ImageJ software to quantify single myofibers’ cross-sectional area (CSA) and count the centrally nucleated fibers. The analysis was performed on at least six transversal sections of each muscle, and a minimum of three fields were randomly chosen.

### Confocal immunofluorescence microscope

Immunofluorescence staining was performed on the tibialis anterior and soleus muscles from *wt* and *arg-ii^-/-^* mice. Briefly, tissues were fixed with 4% paraformaldehyde (pH 7.0) and embedded in paraffin. After deparaffinization in xylene (2 times of 10 minutes), the sections were treated in ethanol (twice in 100% ethanol for 3 minutes and twice in 95% ethanol and once in 80%, 75%, 50% ethanol for 1 minute sequentially) followed by antigen retrieval (tris-EDTA buffer, pH 9.0) for Arg-II (D9J1N, Cell Signaling Technology – 1:50), CD31 (sc-81156, Santa Cruz Biotechnology – 1:100), dystrophin (ab15277, Abcam - 1:100), Vimentin (ab8978, Abcam – 1:100), F4-80 (#30325, Cell Signaling Technology – 1:100), p16^ink^ (sc-81156, Santa Cruz Biotechnology – 1:100), Type-I fibers (BA-F8, DSHB – 1:50), Type-IIA fibers (SC-7, DSHB – 1:50), TGFβ1 (ab215715, Abcam – 1:200), and PAX-7 (Ab528428, DSHB – 1:50) in a pressure cooker. For co-immunofluorescence staining of p16/dystrophin, Arg-II/CD31, Arg-II/Vim, Arg-II/F4-80, and Arg-II/PAX-7, primary antibodies of different species were used. Transverse sections (5 μm) were blocked with mouse Ig blocking reagent (M.O.M, Vector laboratories) for 3 hours and then with PBS containing 1% BSA and 10% goat serum for 1 hour. The sections were then incubated overnight at 4°C in a dark/humidified chamber with target primary antibodies, and subsequently incubated for 2 hours with the following secondary antibodies: Alexa Fluor 488–conjugated goat anti-rabbit IgG (H + L) and Alexa Fluor 568-conjugated goat anti-mouse IgG (H + L); Alexa Fluor 488–conjugated goat anti-mouse IgG (H + L) and Alexa Fluor 594-conjugated goat anti-rabbit IgG (H + L). All sections were finally counterstained with 300 nmol/L DAPI for 5 minutes. Immunofluorescence signals were visualized under the Leica TCS SP5 confocal laser microscope. The antibodies used are shown in the section of Materials and Reagents.

### Muscle fibers typing

The ratio of slow (Type-I) and fast (Type-II) fibers was investigated in the soleus muscles of male and female mice as previously described [48]. Briefly, muscle fiber types were classified according to the distribution of MHC isoforms. In soleus skeletal muscles, types I and IIA were identified by BA-F8 (anti-MHC slow/I) and SC-71 (anti-MHC 2a) respectively. The proportion of each fiber was normalized to the total number of fibers in the selected area, and the ratio of Type-I/Type-II was calculated.

### Masson’s trichrome staining

For these experiments, muscle sections were deparaffined in decreasing gradient of ethanol, fixed in pre-heated Bouin’s solution (ab150686, from Abcam^™^) at 60°C for 1 hour and then at room temperature for 10 minutes, following the manufacturer’s protocol. Sections were washed with water, followed by trichrome staining to discriminate between normal tissue and fibrotic (collagen deposition appeared as a blue stain). Images were acquired through NanoZoomer Hamamatsu. RGB images were split into separate channels using the ImageJ software. The red channel corresponds to the collagen signal and thresholding measurement was performed.

### Quantitative real-time PCR (qRT-PCR)

Total RNA extraction and mRNA expression analysis by 2-step real-time quantitative reverse-transcription polymerase chain reaction (qRT-PCR) were performed. Briefly, total RNA was extracted from the mouse muscle tissues with Trizol Reagent (Molecular Research Center, Inc., Cincinnati, OH, USA) following the supplier’s protocol. The real-time qPCR was performed with the GOTaq^®^ qPCR Master Mix (A6001, Promega) and CFX96 Real-Time PCR Detection System (Bio-Rad, Hercules, CA, USA). The mRNA expression levels of all genes were normalized to the reference gene: glyceraldehyde-3-phosphate dehydrogenase (GAPDH). The mouse-specific primer sequences are listed here in Table 1.

**Table 1:** Primers used for the RT-qPCR.

**Gene name**	**Primer sequence**
*gapdh-F*	5′-ACC CAG AAG ACT GTG GAT GG-3′
*gapdh-R*	5′-ACA CAT TGG GGG TAG GAA CA-3′
*arg-i-F*	5′-GGA ATC TGC ATG GGC AAC CTG TGT-3′
*arg-i-R*	5′-AGG GTC TAC GTC TCG CAA GCC A-3′
*arg-ii-F*	5′-CCC CTT TCT CTC GGG GAC AGA A-3′
*arg-ii-R*	5′-GAA AGG AAA GTG GCT GTC CA-3′
*mcp-1-F*	5′-AGC ACC AGC CAA CTC TCA C-3′
*mcp-1-R*	5′-TCT GGA CCC ATT CCT TCT TG-3′
*il6-F*	5′-GAC AAC CAC GGC CTT CCC TA-3′
*il6-R*	5′-GCC TCC GAC TTG TGA AGT GGT-3′
*tgf-β1-F*	5′-TGG AGC AAC ATG TGG AAC TC-3′
*tgf-β1-R*	5′-CAG CAG CCG GTT ACC AAG-3′
*f4/80-F*	5′-TGG CTG CCT CCC TGA CTT TC-3′
*f4/80-R*	5′-CAA GAT CCC TGC CCT GCA CT-3′
*col1α1-F*	5′-GAT GAC GTG CAA TGC AAT GAA-3′
*col1α1-R*	5′-CCC TCG ACT CCT ACA TCT TCT GA-3′
*col3α1-F*	5′-CAA ACA CGC AAG GCA ATG AGA CTA CC-3′
*col3α- R*	5′-AGG GCC AAT GTC CAC ACC AAA TTC-3′

### Statistical analysis

Statistical analyses were performed with GraphPad Prism Software. All data are expressed as mean ± standard error of the mean (SEM). In all the experiments, “n” indicates the number of independent experiments or the number of mice used in each group. The Shapiro-Wilk test (that determines normality when *n* ≥ 3) was used first to determine whether the data had a normal Gaussian distribution. According to the different types of data, the following statistical analyses are performed. Student’s unpaired *t*-test with a Welch’s correction was used for parametric analyses. Mann–Whitney *t*-tests were used for non-parametric analyses. To analyze the difference between the means of more than two groups normally distributed, an analysis of variance (ANOVA) was performed followed by multiple comparisons with uncorrected Fisher’s least significant difference (LSD) test. If data were not normally distributed, The Kruskal–Wallis test was then applied. For qPCR analysis, each value has been rationalized on the average value of *wt* young groups, giving an “average fold change” value.

### Availability of data and materials

Data supporting the present study are available from the corresponding author upon reasonable request.

## Supplementary Materials

Supplementary Figures
