# Supplementary material for: Arginase-II gene deficiency reduces skeletal muscle aging in mice
Source: Aging (Albany NY). 2024 Dec 12;16(22):13563–87. doi: 10.18632/aging.206173 (PMC11723659; doi:10.18632/aging.206173)
Supplement: Supplementary Figures [file aging-16-206173-s001.pdf]

SUPPLEMENTARY FIGURES

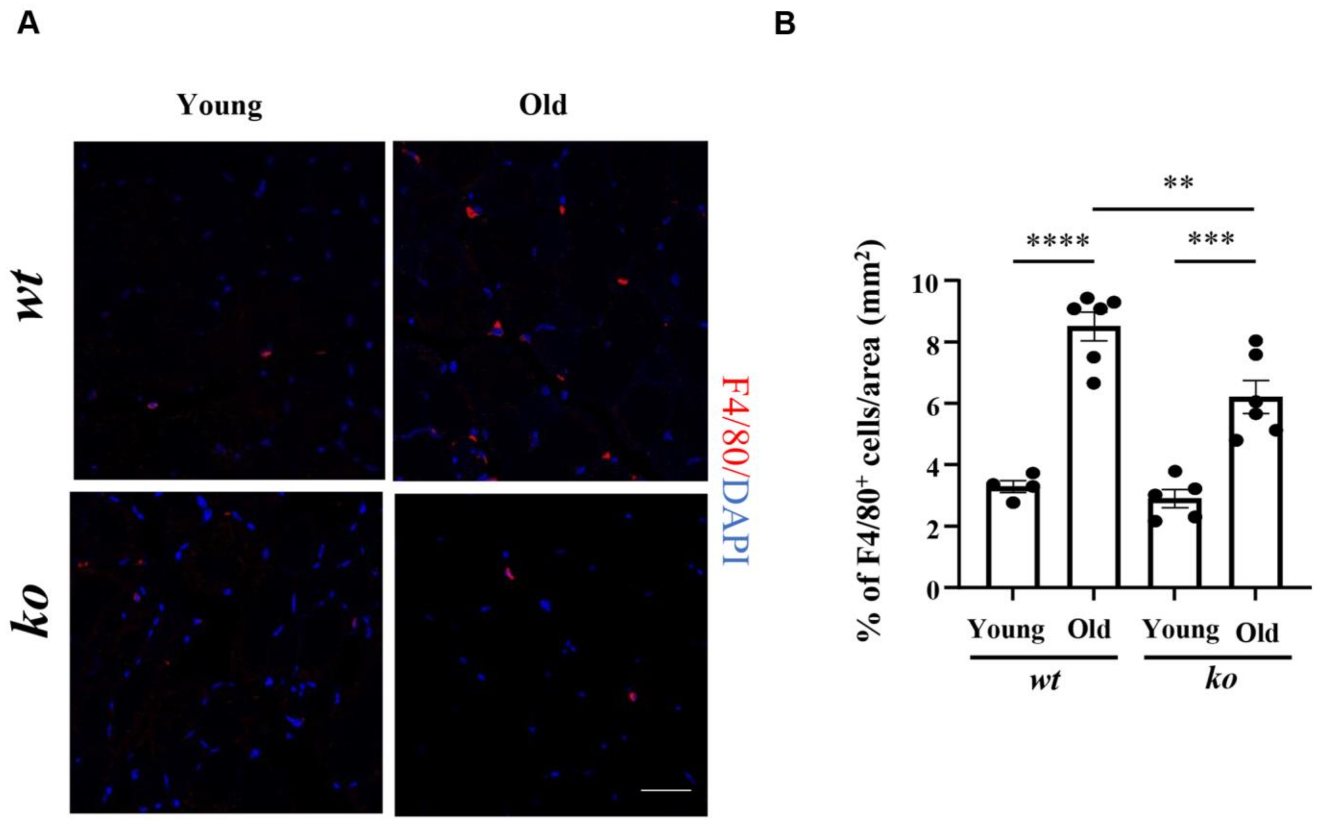

Supplementary Figure 1. *Arg-ii*$^{-/-}$ mice show reduced age-associated F4/80+ macrophages in skeletal muscle fibers. (**A**) representative confocal images of immunofluorescence staining of F4/80+ cells in tibialis of male mice. (**B**) Quantification of F4/80+ cells of the images. *wt, wild type; ko, arg-ii*$^{-/-}$. $^{**}p < 0.01$, $^{***}p < 0.001$, and $^{****}p < 0.0001$ between the groups.

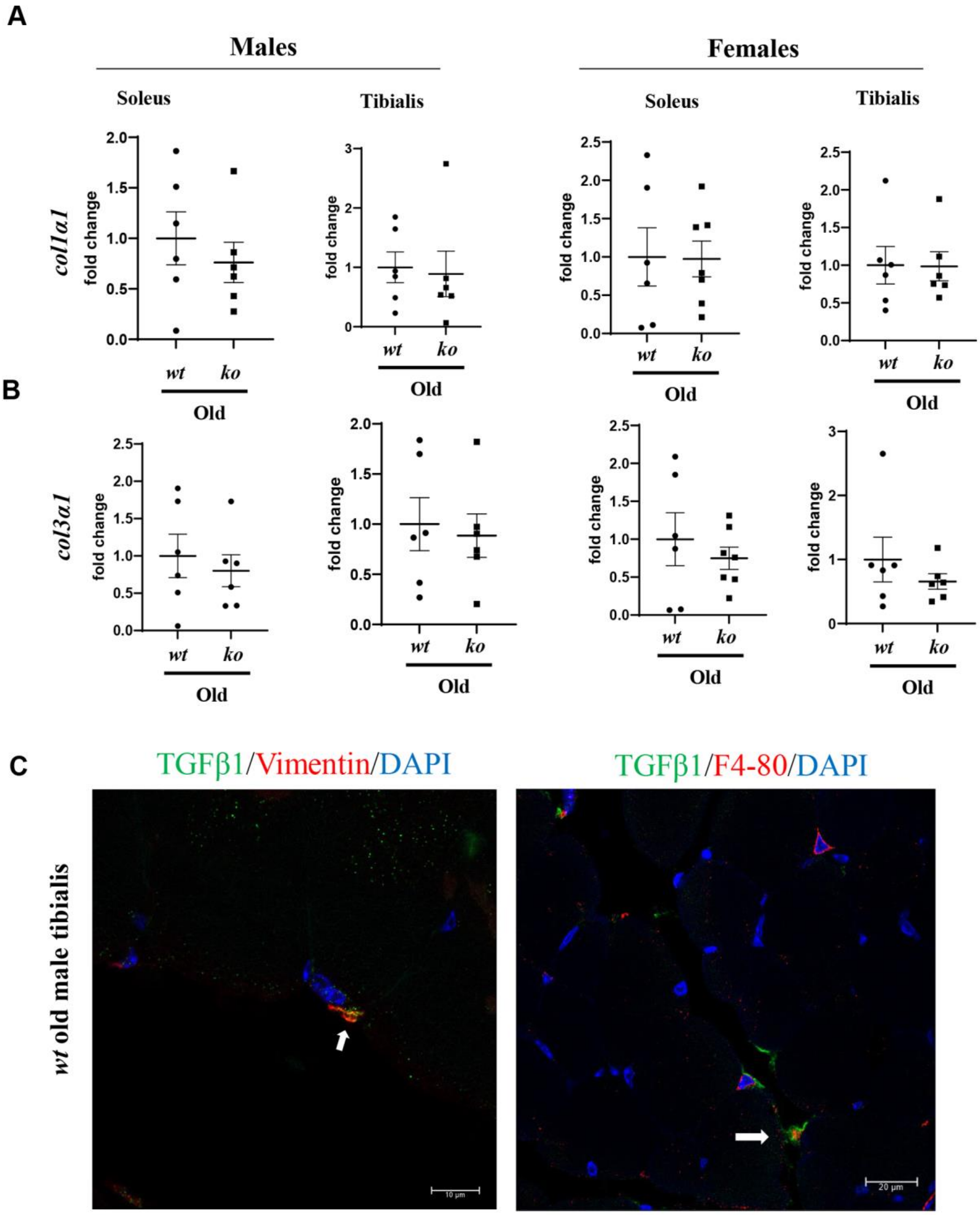

Supplementary Figure 2. Expression profile of collagen genes and cellular localization of TGFb1 in aging skeletal muscle tissues. (**A**, **B**) RT-qPCR analysis of *col1α1* or *col3α1* gene expressions in soleus and tibialis obtained from old mice, *wt:* wild type, *ko:* arg-ii$^{-/-}$. (**C**) Representative images of the tibialis section from male *wt* mice stained with DAPI (blue), anti-*TGFβ1* (green), and anti-vimentin (red, fibroblast marker) or F4/80 (red, macrophage marker). Scale bar: 10 µm.

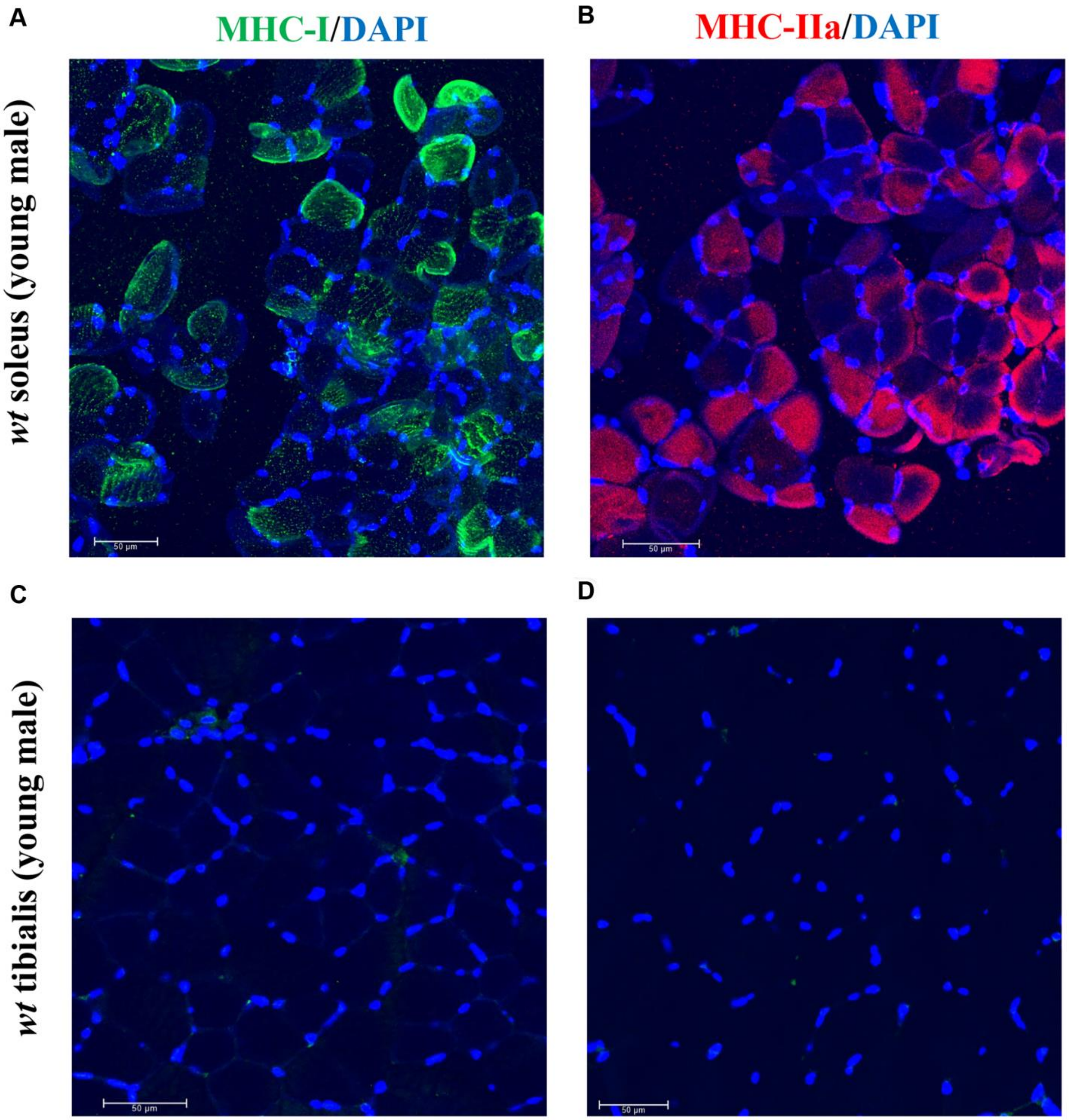

Supplementary Figure 3. Type-I and type-IIa fibers in soleus and tibialis of young male mice. Representative confocal images showing type-I (**A**, **C**) and type-IIa (**B**, **D**) fibers in soleus (**A**, **B**) and tibialis (**C**, **D**) of young *wt* male mice. DAPI (blue) is used to stain nuclei. Scale bar = 50 µm.

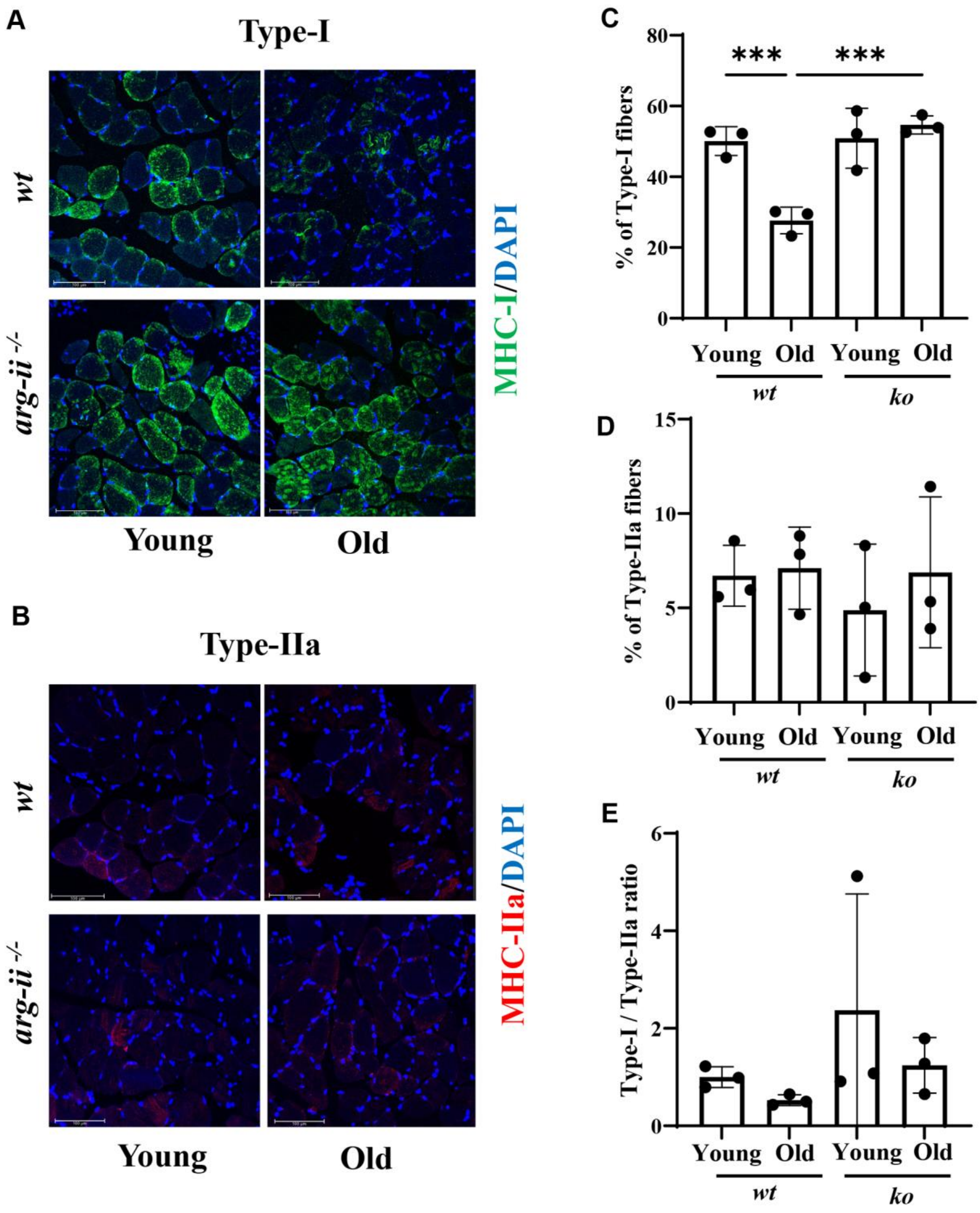

Supplementary Figure 4. *Arg-ii* deficiency prevents age-associated decrease in type-I fibers in female soleus. Representative confocal images showing Type-I (**A**) and Type-IIa (**B**) fibers in soleus of young and old *wt* and *arg-ii*$^{-/-}$ (*ko*) female mice. DAPI (blue) is used to stain nuclei. Scale bar = 100 µm. The graphs present the percentage of Type-I (**C**) and Type-II (**D**) fibers considering the total number of fibers in the image. (**E**) the graph shows the ratio of Type-I and Type-II fibers expressed as fold change (*wt* young group was taken as reference. One-way ANOVA test was applied. *n* = 3 mice per group. *wt, wild type; ko,arg-ii*$^{-/-}$. ***p < 0.001 between the indicated groups.

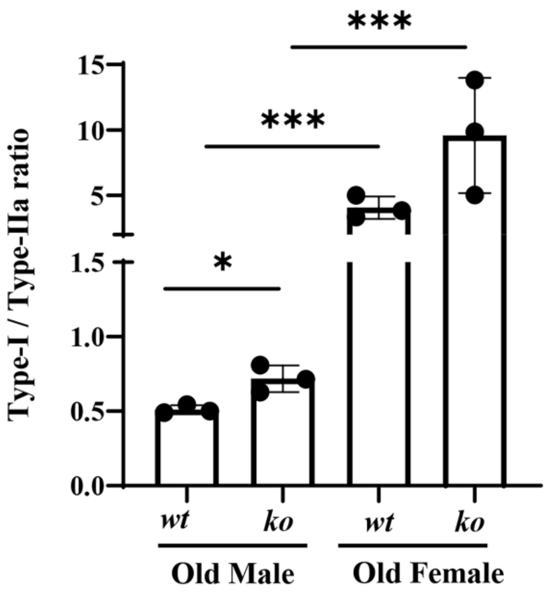

Supplementary Figure 5. Type-I/Type-IIa ratio the graph shows the ratio of Type-I and Type-II fibers in old *wt* and *arg-ii*$^{-/-}$ (*ko*) mice of both sexes. One-way ANOVA test was applied. *n* = 3 mice per group. *wt, wild type; ko, arg-ii*$^{-/-}$. $^{***}p < 0.001$ between the indicated groups.
